# Impact of coronavirus outbreak on psychological health

**DOI:** 10.7189/jogh.10.010331

**Published:** 2020-06

**Authors:** Suliman Khan, Rabeea Siddique, Hongmin Li, Ashaq Ali, Muhammad Adnan Shereen, Nadia Bashir, Mengzhou Xue

**Affiliations:** 1 The Department of Cerebrovascular Diseases, The Second Affiliated Hospital of Zhengzhou University, Zhengzhou, Henan, China; 2 Henan Medical Key Laboratory of Translational Cerebrovascular Diseases, Zhengzhou, Henan, China; 3 Wuhan Institute of Virology, Chinese Academy of Sciences Xiao Hong Shan No.44, Wuhan, Hubei, China; 4 State Key Laboratory of Virology, College of Life Sciences, Wuhan University, Wuhan, Hubei, China

**Figure:**
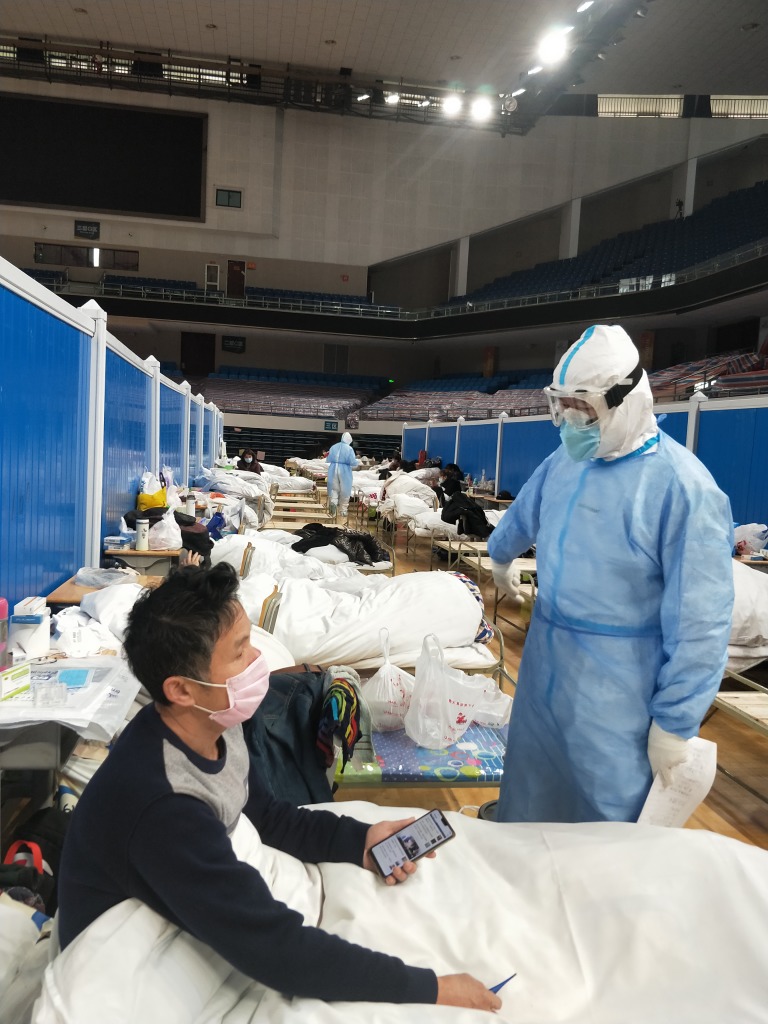
Photo: Chinese patients with COVID-19 are provided with mental health counseling during the COVID-19 epidemic in Wuhan, Hubei, China (from the collection of Suliman Khan, used with permission).

The emergence of novel coronavirus called severe respiratory syndrome coronavirus 2 (SARS-CoV-2) caused a deadly pneumonia outbreak in Wuhan, China [1,2]. It is thought to be the continuation of serious epidemics caused by Middle East respiratory syndrome coronavirus (MERS-CoV) and severe acute respiratory syndrome (SARS) in Middle East (2009) and Guangdong, China (2002-03), respectively [3-6]. The COVID-19 infection, soon after its emergence not only caused a number of deaths in China but rapidly spread to other countries as well [7]. The emergence of SARS-CoV-2 and the menace of imminent endemics have brought into forefront the urgent need to prepare for the consequences of associated epidemics and pandemics. The aftermath of such outbreaks not only harms physical but mental health as well, thus it is necessary to identify mental health abnormalities and to properly utilize effective therapies (**Figure 1**).

**Figure 1:**
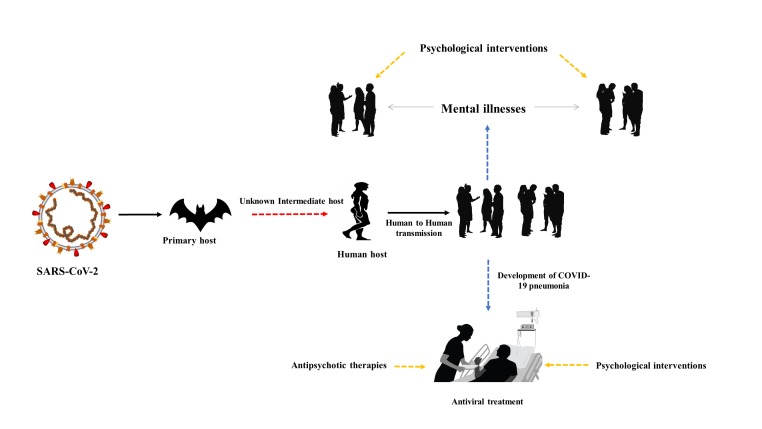
Psychological interventions.

Currently, primary importance is given to the physical health that includes therapies and treatment to pneumonic symptoms. Hence, ignoring the profound importance of psychological health, ensued by the viral infection, isolation, restricted social activities, troubled sleeping, lockdown and forged news; culminating into stress, anxiety, and episodes of depressive reactions. Not only epidemic but also infodemic poses serious problems for public health [8] that may further increase the risk of mental illnesses. In this paper, we focus on the risks of psychiatric disorders associated with novel coronavirus outbreak, and its effective communication to build psychological resilience among emergency health workers and the public. Then we summarize some facts acquired in SARS and MERS outbreaks to assist the vulnerable populations in coping with psychiatric disorders. We further discuss the consequences of being in stress, and interventions and medications for ameliorating stress and related psychiatric conditions to improve psychological health.

## OUTBREAK’S IMPACT ON PEOPLE AND THEIR RESPONSES

In a broad aspect of viral outbreaks, it is impossible to predict the extent of mortality and morbidity accurately, that will be caused by a newly emerged pathogenic virus. Considering the current situation of the world environment, viral outbreaks may affect animals or humans [3,9], without causing extensive fatalities, however, their psychological impacts can be serious such as anxiety, insomnia, panic behavior, fear, and hopelessness. In some cases viral outbreaks infect thousands of people, cause hundreds to thousands of fatalities and spread around the globe [1,3,10,11], thereby affecting millions of people to induce anxiety, panic behavior, and other related psychiatric disorders. To cope with such outbreaks and epidemics, the health care authorities should have affective plans, which must consider psychological health [3,10,12]. Unlike the infected individuals, the responses of uninfected individuals are expected to be mild, however, these reactions become worse by rumors spread by media and social networks [13-15]. In the outbreak caused by 2019-nCoV in Wuhan, China, the reactions of the people could be quite severe as compared to the outbreaks of SARS and MERS [3,11] (and references S1-S3 in the **Online Supplementary Document**; other supplementary references are indicated by letter S in brackets).

Interventions by health care authorities have consisted of equivocal recommendations for the prevention of viral infections (S4). For instance, some of the infected individuals were advised to stay isolated inside homes rather than hospitals, which may increase the stress burden on family members due to the fear of getting infected. The scarcity of preventive measurements such as gloves, masks, and headcovers during the massive lockdown in China may induce further stress in the general population. To mitigate the negative outcomes, interventions for the general population must be informed by these recommendations; information about viral outbreak and possible impact, individual risk, outcomes of negative health behaviors, identifying place and time of risk in advance to take specific measurements, public awareness regarding necessary actions to prevent the spread of viral infections. Furthermore, people must be provided with follow-up information during and after control of the outbreak.

## IMPACT OF INFODEMIC ON PUBLIC HEALTH

The term infodemia was coined during the SARS outbreak, however, it is becoming comparatively more serious in the outbreak of COVID-19 infection. This term is used to present the epidemic of rapidly spreading misinformation through social media platforms and other outlets [8]. Winning the race of the sharing novel details for COVID-19 and gaining fame over social media have accelerated the rate of spreading forged news during the current outbreak of COVID-19. This situation is leading to cause massive infodemic, which may increase the risk of severe public health consequences. Aiming at sharing tailored information with specific target groups using a series of amplifiers, WHO’s risk communication team launched a new information platform known as WHO Information Network for Epidemics. This platform actively reported the details related to COVID-19 after COVID-19 was declared a Public Health Emergency of International Concern [8]. However, a large population is either not aware or has no access to the information provided. Therefore, people who use social networks excessively are prone to adverse effects related to infodemic. The major concerns associated with infodemic can further add to the psychological stress and anxiety. To mitigate the risks associated with infodemic the public health community should provide help to social and conventional media to better understand the actual concerns associated with the outbreak and positively portray the efforts from health care services providers, medical associations and scientific communities.

## MEASUREMENTS AND RESPONSES

### Resilience of affected and/or infected individuals

Psychological resilience is generally defined as “the ability to sustain or recuperate, psychological well-being during or after facing the stressful conditions” (S5). In the current scenario of the COVID-19 outbreak in Wuhan, China, resilience can be achieved by ending the vulnerability to psychopathology and mental dysfunction when exposed to the viral infection or at-least living in the regions under threat. People of Wuhan may experience unusual stress, anxiety, and even serious mental health issues in response to minor stressors that people during normal conditions would cope with readily (S5-S8). Resilience is mediated by the presence of distinct molecular adaptations to promote normal behavioral function and the absence of molecular abnormalities that occur in susceptible individuals that impairs the coping ability (S6). Thus, prevention from the stress and mental impairment requires building resilience in people as a whole. Different strategies including biological, social and psychological mechanisms are associated with increasing resilience (S5-S9). While combating the impact of COVID-19 pneumonia (**Figure 1**), the health care regulatory authorities should communicate the people that the risk of being infected may sustain for a long time, but the government will protect the nation. In addition, it should further be communicated that serious measurements will be implemented at hospitals and communities. Moreover, people should be given a hope that everything will become normal and controlled with the passage of time. The scientists and health care workers should communicate the affected population about their progress regarding developing treatment and prevention strategies and improving facilities.

### Regions under secondary threat

The regions and areas other than the originating region of COVID-19 infection will be considered as the regions under secondary threat. In these regions, the stress and fear of infection along with other above-mentioned factors will likely generate moderate to major behavioral and psychological abnormalities (S10-S12). The overall impact of infection on mental health will be associated with the rate of infectiousness and mortality.

The most common impacts of such an outbreak or epidemic could be anxiety, panic behavior, sleep disturbances, disrupted daily biological rhythms, anger, and disappointment (S10,S13-S15). In most cases, infected people make their utmost effort to avoid the spread of the virus in community however, some people don’t cooperate. It has been generally observed in several cities of China that foreigners (mostly students) find ways to flee. It may be due to the higher stress or fear; however, such actions may cause the viral spread in countries with no facilities for combating coronavirus. It will ultimately increase the burden of mental abnormalities in the respective population. To avoid the negative outcomes of such practices, the relevant forces should communicate people who can help them identify such individuals. It may be possible through facilitating the community by electronic and social media. Individuals who have moderate psychological disturbances can be handled by mental health professionals through relaxation and cognitive behavioral techniques, and/or medication if needed (S11,S16). Furthermore, people must be educated or made aware of the development of clinical depression, and suicidality related symptoms followed by the outbreak. Professional clinical psychologists or psychiatrists should come forward and communicate through media to encourage individuals for life and healthy activities. In addition, communities with certain beliefs should be benefited from spiritual education. Nevertheless, media can play a critical role by broadcasting instruction regarding relaxation, meditation, and advice to not focus on rumors and forged news from unauthentic sources.

### Regions under primary threat

The area of novel coronavirus origination (Wuhan) will be considered as the region under the primary threat. The outbreak of COVID-19 pneumonia imposed a serious threat to the residents of the city by infecting thousands of people and killing hundreds of them [11] (S2,S4,S7). Similar outbreaks were observed in past caused by the coronaviruses namely, SARS-coronavirus and MERS-CoV, however the transmission rate in those cases was significantly lower as compared to 2019-nCoV of Wuhan [3,10,11] (S17). MERS negatively impacted mental health up to a higher extent and in some cases, the impacts imposed were long term and severe (S18-S22). Comparatively, the COVID-19 may have strong negative effects on health and can cause serious mental problems including acute stress, insomnia, severe anxiety, and chronic depression. MERS and SARS induced serious psychological problems majorly in health care workers (S22), however, the COVID-19 infection may cause such abnormalities in the general public as well, because it is more intense in terms of infecting and killing the individuals [11]. To overcome serious problems in the future, all of the recommendations that we have discussed in the previous section of regions at secondary risks should be implemented here. Furthermore, the people should be provided with counseling and guidance at specified public centers, equipped with psychologists, and psychiatrists.

## THERAPEUTIC OR RECOVERY STRATEGIES

For the first time in the world’s history, a whole city is on medical quarantine, with peak fear and havoc in Wuhan after the COVID-19 outbreak [1,11] (S3). In addition to serious mortality, rapid transmission and higher morbidity are increasing hysteria and anxiety among people. People in Wuhan are under critical conditions that may increase panic behavior. People are scared of being infected by their fellows and even family members, thus, they prefer to remain isolated and locked down, while overusing the electronic devices. For foreigners, the conditions are more severe as countries have started evacuating some of the people and leaving others under the burden of stress. Thus, a proper system must be developed to educate people through psychological counseling. Moreover, they should be provided with pharmacological therapies and psychiatric assistance where needed.

### Psychological counseling based therapies

The alleviation of acute distress and preventing chronic depression, anxiety and posttraumatic stress among people and health care workers indirectly exposed to the carnage of viral infection. Psychological resilience will be important in the population who are likely to develop psychotic or traumatic symptoms irrespective of their severity. Furthermore, people with acute stress disorder or depressive disorders will also be dealt with psychological debriefing by administering briefing sessions (S22,S23) in which the participants are asked to describe their feelings and behavioral reactions during the epidemic. The instructors provide psychoeducation by teaching that such psychological problems are normal responses to such outbreaks (S23). In addition, cognitive behavior therapy may also effective in combating the psychological and psychiatric symptoms followed by the 2019-nCoV outbreak in Wuhan, China (S24,S25). Exposure based therapy may also be useful in the scenario of current situation. In this therapy exposure to some good memories and events is made. Moreover, stress inoculation training for depression, stress, and related disorders, education about these disorders related symptoms and techniques to manage anxiety including relaxation training, guided self-dialogue and cognitive restructuring (S24-S26). In addition, cognitive therapy may also be helpful in which the affected person is taught to identify irrational or dysfunctional beliefs about symptoms and to challenge these beliefs logically (S25-S26). Interpersonal therapy can also be selected, which focuses on interpersonal relationships (S27).

### Pharmacological therapies

Pharmacological treatment of depression, anxiety, and related disorders is an alternate or in some cases adjunct to psychological treatment. Drugs that influence glutamatergic, adrenergic, serotonergic, endocannabinoid systems and various neuropeptide are generally preferred. In such therapies antidepressants are the first-line pharmacological treatment options such as serotonin-noradrenaline reuptake inhibitors (SNRIs) and selective serotonin reuptake inhibitors (SSRIs). These drugs are effective against several types of mental disorders specifically depression and anxiety (S28-S30). Agomelatine (a melatonin receptor agonist) and Vilazodone (a serotonin reuptake inhibitor and partial agonist of serotonin receptors) are also recommended in depression (S27,S31). Among second-line agents for anxiety and depressive disorders benzodiazepines have been found with good results. However, they have some serious adverse effects including dizziness, drowsiness, and increased risk of falls. In such cases the alternative drugs (that modulate GABA signaling), including, gabapentin and pregabalin can be used. In case of severe anxiety, risperidone or quetiapine (atypical antipsychotics) should be given as an adjunct to SSRIs or SNRIs (S27,S31-S33). All these drugs could be effective; however, their use should be only recommended when psychological approaches are not working. Nevertheless, the use of both pharmacotherapy and psychotherapy (combined therapy) is more beneficial and effective in the case of chronic anxiety and depressive disorders (S27).

## SUMMARY AND CONCLUSION

In the view of continued viral outbreaks around the world and specifically in China that affects millions of people, it is imperative to evaluate and develop strategies to address psychological health and psychiatric aberrations caused by direct or indirect exposure to the situation. These strategies are specific to target the communities or entire populations as well as the individuals with psychiatric symptoms resulting from the actions taken by the government against coronavirus epidemic, viral infection, and fear of infection. Generally targeting the entire population or large communities is not beneficial thus targeting individual-based therapies should be given priority. Based on its experience at a wide scale in the past, psychological resilience can be an effective strategy during the days of epidemic or outbreak. However, cognition-based therapy will be effective after the epidemic is ended. Generally, psychological debriefing is recommended for the people who gain stress immediately while brief cognitive-behavior therapy is recommended for people with severe stress symptoms few weeks after the incident. This therapeutic strategy should be preferred for the individuals directly exposed to the epidemic and directly faced the preventive measurements imposed by the government, i.e, the residents of Wuhan. These individuals are experiencing high levels of stress and are vulnerable to develop serious mental abnormalities. Besides these approaches, other psychological treatments should also be evaluated and employed. There are possibilities that a large population develops severe psychiatric symptoms by disruption of molecular pathways, thus, the administration of medication is recommended. Overall, the individuals who are going through the stress and prone to develop serious symptoms of psychiatric disorders in later stages must be addressed properly. In addition to the general population exposed to the viral outbreak, health care workers need serious attention and psychological counseling.

## Additional material

Online Supplementary Document

## References

[1] Huang C Wang Y Li X Ren L Zhao J Hu Y Clinical features of patients infected with 2019 novel coronavirus in Wuhan, China. Lancet. 2020;395:497-506. 10.1016/S0140-6736(20)30183-5 31986264 PMC7159299

[2] Khan S Ali A Siddique R Nabi G Novel coronavirus is putting the whole world on alert. J Hosp Infect. 2020;104:252-3. 10.1016/j.jhin.2020.01.019 32032614 PMC7134434

[3] Cui J Li F Shi ZL Origin and evolution of pathogenic coronaviruses. Nat Rev Microbiol. 2019;17:181-92. 10.1038/s41579-018-0118-9 30531947 PMC7097006

[4] Zhong NS Zheng BJ Li YM Poon LLM Xie ZH Chan KH Epidemiology and cause of severe acute respiratory syndrome (SARS) in Guangdong, People’s Republic of China, in February 2003. Lancet. 2003;362:1353-8. 10.1016/S0140-6736(03)14630-2 14585636 PMC7112415

[5] Bawazir A Al-Mazroo E Jradi H Ahmed A Badri M MERS-CoV infection: Mind the public knowledge gap. J Infect Public Health. 2018;11:89-93. 10.1016/j.jiph.2017.05.003 28647126 PMC7102865

[6] Zaki AM Van Boheemen S Bestebroer TM Osterhaus ADME Fouchier RAM Isolation of a novel coronavirus from a man with pneumonia in Saudi Arabia. N Engl J Med. 2012;367:1814-20. 10.1056/NEJMoa1211721 23075143

[7] World Health Organization. Coronavirus disease 2019 (COVID-19) situation report. February 28, 2020. Geneva: WHO; 2020.

[8] Zarocostas J How to fight an infodemic. Lancet. 2020;395:676. 10.1016/S0140-6736(20)30461-X 32113495 PMC7133615

[9] Zhou P Fan H Lan T Yang X-L Shi W-F Zhang W Fatal swine acute diarrhoea syndrome caused by an HKU2-related coronavirus of bat origin. Nature. 2018;556:255-8. 10.1038/s41586-018-0010-9 29618817 PMC7094983

[10] Liu S Chan TC Chu YT Wu JTS Geng X Zhao N Comparative epidemiology of human infections with middle east respiratory syndrome and severe acute respiratory syndrome coronaviruses among healthcare personnel. PLoS One. 2016;11:e0149988. 10.1371/journal.pone.0149988 26930074 PMC4773072

[11] Hui DSI Azhar E Madani TA Ntoumi F Kock R Dar O The continuing 2019-nCoV epidemic threat of novel coronaviruses to global health — The latest 2019 novel coronavirus outbreak in Wuhan, China. Int J Infect Dis. 2020;91:264-6. 10.1016/j.ijid.2020.01.009 31953166 PMC7128332

[12] Khan S Siddique R Shereen MA Ali A Liu J Bai Q The emergence of a novel coronavirus (SARS-CoV-2), their biology and therapeutic options. J Clin Microbiol. 2020; JCM.00187-20 [Epub ahead of print]. 10.1128/JCM.00187-20 32161092 PMC7180238

[13] Escobar-Viera CG Whitfield DL Wessel CB Shensa A Sidani JE Brown AL For better or for worse? A systematic review of the evidence on social media use and depression among lesbian, gay, and bisexual minorities. JMIR Ment Health. 2018;5:e10496. 10.2196/10496 30037786 PMC6079300

[14] Ruzek JI Eric Kuhn E Jaworski BK Owen JE Ramsey KM Mobile mental health interventions following war and disaster. mHealth. 2016;2:37. 10.21037/mhealth.2016.08.06 28293610 PMC5344166

[15] Jones NM Thompson RR Schetter CD Silver RC Distress and rumor exposure on social media during a campus lockdown. Proc Natl Acad Sci U S A. 2017;114:11663-8. 10.1073/pnas.1708518114 29042513 PMC5676907

