# Supplementary material for: Impact of coronavirus outbreak on psychological health
Source: J Glob Health. 2020 Apr 9;10(1):010331. doi: 10.7189/jogh.10.010331 (PMC7180007; doi:10.7189/jogh.10.010331)
Supplement: Online Supplementary Document [file jogh-10-010331-s001.pdf]

S1. Totura AL, Bavari S. Broad-spectrum coronavirus antiviral drug discovery. Expert Opin Drug Discov . 2019;14(4):397–412.

S2. Khan S, Nabi G, Han G, Siddique R, Lian S, Shi H, et al. Novel coronavirus: how the things are in Wuhan. Clin Microbiol Infect . 2020.

S3. Khan S, Siddique R, Ali A, Xue M, Nabi G. Novel coronavirus, poor quarantine, and the risk of pandemic. J Hosp Infect . 2020.

S4. Rapid outbreak response requires trust. Nat Microbiol. 2020;5,227–228.

S5. Laird KT, Krause B, Funes C, Lavretsky H. Psychobiological factors of resilience and depression in late life. Transl Psychiatry . 2019;9(1).

S6. Russo SJ, Murrough JW, Han MH, Charney DS, Nestler EJ. Neurobiology of resilience. Nat Neurosci . 2012;15(11):1475–84.

S7. Bonde JPE. Psychosocial factors at work and risk of depression: A systematic review of the epidemiological evidence. Occup Environ Med. 2008;65(7):438–45.

S8. Gómez-Lázaro E, Garmendia L, Beitia G, Perez-Tejada J, Azpiroz A, Arregi A. Effects of a putative antidepressant with a rapid onset of action in defeated mice with different coping strategies. Prog Neuro-Psychopharmacology Biol Psychiatry . 2012;38(2):317–27.

S9. Feder A, Nestler EJ, Charney DS. Psychobiology and molecular genetics of resilience. Nat Rev Neurosci . 2009;10(6):446–57.

S10. Aaby A, Ravn SL, Kasch H, Andersen TE. The associations of acceptance with quality of life and mental health following spinal cord injury: a systematic review. Spinal Cord . 2019.

S11. Evers AWM, Zautra A, Thieme K. Stress and resilience in rheumatic diseases: A review and glimpse into the future. Nat Rev Rheumatol . 2011;7(7):409–15.

S12. Munshi S, Loh MK, Ferrara N, Dejoseph MR, Ritger A, Padival M, et al. Repeated Stress Induces a Pro-inflammatory State, Increases Amygdala Neuronal and Microglial Activation, and Causes Anxiety in Adult Male Rats. Brain Behav Immun . 2019.

S13. Halperin D. Environmental noise and sleep disturbances: A threat to health? Sleep Sci . 2014;7(4):209–12.

S14. Mcclung CA. Circadian rhythms and mood regulation: Insights from pre-clinical models. Eur Neuropsychopharmacol . 2011;21(SUPPL.4):S683–93.

S15. Hackett RA, Steptoe A. Type 2 diabetes mellitus and psychological stress-a modifiable risk factor. Nat Rev Endocrinol . 2017;13(9):547–60.

S16. Laraque D, Boscarino JA, Battista A, Fleischman A, Casalino M, Hu Y, et al.

September 11th : Implications for Children ’ s Mental Health Services. Pediatrics. 2004;113(5):1357–66.

S17. Shereen MA, Khan S, Kazmi A, Bashir N, Siddique R. COVID-19 infection: origin, transmission, and characteristics of human coronaviruses. J Adv Res. 2020.

S18. Lee SM, Kang WS, Cho AR, Kim T, Park JK. Psychological impact of the 2015 MERS outbreak on hospital workers and quarantined hemodialysis patients. Compr Psychiatry . 2018;87:123–7.

S19. Almutairi AF, Adlan AA, Balkhy HH, Abbas OA, Clark AM. “It feels like I’m the dirtiest person in the world.”: Exploring the experiences of healthcare providers who survived MERS-CoV in Saudi Arabia. J Infect Public Health . 2018;11(2):187–91.

S20. Bukhari EE, Temsah MH, Aleyadhy AA, Alrabiaa AA, Alhboob AA, Jamal AA, et al. Middle east respiratory syndrome coronavirus (MERS-CoV) outbreak perceptions of risk and stress evaluation in nurses. J Infect Dev Ctries. 2016;10(8):845–50.

S21. Khalid I, Khalid TJ, Qabajah MR, Barnard AG, Qushmaq IA. Healthcare workers emotions, perceived stressors and coping strategies during a MERS-CoV outbreak. Clin Med Res. 2016;14(1):7–14.

S22. Roberts NP, Kitchiner NJ, Kenardy J, Robertson L, Lewis C, Bisson JI. Multiple session early psychological interventions for the prevention of post-traumatic stress disorder. Cochrane Database Syst Rev. 2019;2019(8).

S23. Harder N, Lemoine J, Harwood R. Psychological outcomes of debriefing healthcare providers who experience expected and unexpected patient death in clinical or simulation experiences: A scoping review. J Clin Nurs. 2020;29(3–4):330–46.

S24. Duffy D, Enrique A, Connell S, Connolly C, Richards D. Internet-Delivered Cognitive Behavior Therapy as a Prequel to Face-To-Face Therapy for Depression and Anxiety: A Naturalistic Observation. Front Psychiatry. 2020;10(January):1–15.

S25. Campbell-Sills L, Roy-Byrne PP, Craske MG, Bystritsky A, Sullivan G, Stein MB. Improving Outcomes for Patients With Medication-Resistant Anxiety: Effects of Collaborative Care With Cognitive Behavioral Therapy. Focus. 2017;15(2):211–8.

S26. Santiago J, Akeman E, Kirlic N, Clausen AN, Cosgrove KT, McDermott TJ, et al. Protocol for a randomized controlled trial examining multilevel prediction of response to behavioral activation and exposure-based therapy for generalized anxiety disorder. Trials. 2020;21(1):1–14.

S27. Craske MG, Stein MB, Eley TC, Milad MR, Holmes A, Rapee RM, et al. Anxiety disorders. Nat Rev Dis Prim. 2017;3.

S28. Miller V. Anxiety disorders. Vol. 40, Australian Family Physician. 2011. 755.

S29. Warner-Schmidt JL, Vanover KE, Chen EY, Marshall JJ, Greengard P. Antidepressant effects of selective serotonin reuptake inhibitors (SSRIs) are attenuated by antiinflammatory drugs in mice and humans. Proc Natl Acad Sci. 2011;108(22):9262–7.

S30. Wang Y, Liu X, Yu Y, Han Y, Wei J, Collier D, et al. The role of single nucleotide polymorphism of D2dopamine receptor gene on major depressive disorder and response to antidepressant treatment. Psychiatry Res . 2012;200(2–3):1047–50.

S31. David DJP, Bourin M, Jego G, Przybylski C, Jolliet P, Gardier AM. Effects of acute treatment with paroxetine, citalopram and venlafaxine in vivo on noradrenaline and serotonin outflow: A microdialysis study in Swiss mice. Br J Pharmacol. 2003;140(6):1128–36.

S32. Li Y, Zhong W, Wang D, Feng Q, Liu Z, Zhou J, et al. Serotonin neurons in the dorsal raphe nucleus encode reward signals. Nat Commun. 2016;7.

S33. Lucas G, Rymar V V., Du J, Mnie-Filali O, Bisgaard C, Manta S, et al. Serotonin4(5-HT4) Receptor Agonists Are Putative Antidepressants with a Rapid Onset of Action. Neuron. 2007;55(5):712–25.
